# Social Context Influences Aggressive and Courtship Behavior in a Cichlid Fish

**DOI:** 10.1371/journal.pone.0032781

**Published:** 2012-07-12

**Authors:** Julie K. Desjardins, Hans A. Hofmann, Russell D. Fernald

**Affiliations:** 1 Department of Biology, Stanford University, Stanford, California, United States of America; 2 Section of Integrative Biology, Institute for Cellular and Molecular Biology, Institute for Neuroscience, The University of Texas at Austin, Austin, Texas, United States of America; Cajal Institute, Consejo Superior de Investigaciones Científicas, Spain

## Abstract

Social interactions require knowledge of the environment and status of others, which can be acquired indirectly by observing the behavior of others. When being observed, animals can also alter their signals based on who is watching. Here we observed how male cichlid fish (*Astatotilapia burtoni)* behave when being watched in two different contexts. In the first, we show that aggressive and courtship behaviors displayed by subordinate males depends critically on whether dominant males can see them, and in the second, we manipulated who was watching aggressive interactions and showed that dominant males will change their behavior depending on audience composition. In both cases, when a more dominant individual is out of view and the audience consists of more subordinate individuals, those males signal key social information to females by displaying courtship and dominant behaviors. In contrast, when a dominant male is present, males cease both aggression and courtship. These data suggest that males are keenly aware of their social environment and modulate their aggressive and courtship behaviors strategically for reproductive and social advantage.

## Introduction

Within group living or colonial animals, to be successful individuals need to know specific details about their environment and their status relative to other individuals. Animals gain such information either directly through interactions, or indirectly through observation. Animal signals in this context can be directed at an intended receiver but also seen by bystanders in the social community (e.g., [1]). Moreover, animal signals can change when an individual is being observed in some cases possibly intended, and such changes may differ depending on the identity of the observers [2], [3], [4], [5], [6].

In animal groups, in addition to individual interactions between individuals, attention hierarchies often exist in social groups where more subordinate individuals monitor the behavior more dominant or higher ranking individuals [7]. For example, human children are acutely aware of social hierarchies and modulate their aggressive behavior based on the presence or absence of a more dominant individual [8]. Specifically, when a more dominant individual aggresses upon a subordinate child, that individual, in turn becomes aggressive towards another individual more subordinate than themselves [9]. This attention structure and displaced aggression has been documented in a number of vertebrate species including baboons [10], reptiles [11], trout [12], and can have profound effects on individual health [13].

To understand the effects of social context on behavior, we have analyzed behavioral interactions in a highly social animal, the African cichlid fish, *Astatotilapia burtoni.* In males of this species, reproductive capacity is tightly linked to social status [14] making it an ideal model to measure the influence of a social group on aggression and discover whether there is an attention hierarchy. Dominant or territorial (T) males have bright coloration, defend a spawning territory and display aggressive and courtship behaviors. Subordinate or non-territorial (NT) males are drably colored, do not occupy or defend any spawning territories and typically school with females [15]. When a subordinate male perceives a social opportunity to ascend in status and become dominant, he displays territorial and reproductive behavior within minutes. These males also show evidence of rapid activation at all stages of the hypothalamus-pituitary-gonad axis [16], [17]. This allows them to quickly (<3 days) become reproductively competent. Part of decision to attempt to ascend in social status depends on recognizing the relative strength of possible opponents. *A. burtoni* males can construct a hierarchy amongst other males from observation of fights between conspecifics using transitive inference [18].

To better understand how social information is collected and used, we asked two questions: 1) Is there an attention hierarchy in social groups of *A. burtoni?* And 2) How does the presence of an audience influence fighting in *A. burtoni?* The first question focused on whether fish are attending and to whom in the dominance hierarchy, and the second question tested whether the kind of observers influences aggressive behavior. We conducted two experiments, first recording fish in naturalistic communities and then manipulating group composition to quantify differences in aggression.

## Methods

### Ethical Statement

This study was performed in strict accordance with the recommendations in the Stanford's Administrative Panel for Laboratory Animal Care (APLAC) and this study was specifically approved by Stanford University's APLAC board (protocol #9882). All aggressive encounters were closely monitored and would have been stopped in the event of injury in an effort to minimize suffering.

### General methods

*A. burtoni* males were kept in aquaria under conditions mimicking their natural environment ([15]: pH 8, 28°C water temperature and 12∶12 L:D full spectrum lighting). Gravel covered the floor of the main housing aquaria (121cm L×45 cm W×25 cm H), and all tanks were equipped with half clay flowerpot shelters that the animals excavated and allowed for the establishment and maintenance of territories necessary for successful reproduction [19]. When in the flowerpot shelters, animals are out of view of other fish and vice-versa. Fish were fed each morning with cichlid pellets and flakes (AquaDine, Healdsburg, CA).

### Part 1 – Examining attention hierarchies

To discover whether there is an attention hierarchy in *A. burtoni*, social groups comprised of ten males and ten females were marked with unique combinations of colored beads attached near the dorsal fin and placed in an aquarium (identical in dimensions to the ones described above) that was isolated from outside stimulus using black felt cloth. This isolation was necessary to document social interactions within the social group and not interactions taking place between social groups through adjacent aquaria. After an acclimation period of approximately 1 week, behavior was videotaped (Sony DCR-TRV900, NTSC) from directly overhead for one hour each day between 10∶00 and 15∶00 for two weeks. The one-week acclimation period was necessary for the fish to establish a dominance hierarchy and behave normally. Individual male behaviors were quantified from video playbacks and categorized according to criteria from Fernald [19]: Aggressive behaviors: chasing males, chasing females, threat displays, border conflicts and carousels and non-aggressive behaviors: fleeing and courtship behaviors: courting and leading females. Individual males were characterized as either T (dominant) or NT (Subordinate). Dominant males were easily identified because they express bright blue or yellow body coloration and display a dramatic black lachrymal stripe across the eyes and non-dominant males are cryptically colored and do not express the lachrymal stripe (for review see [14]). In addition, we measured when dominant males entered and exited their shelters relative to the visual line of sight between observed dominant fish and the subordinate animals to determine whether or not the dominant male could be seen or not by subordinate males. This part of the experiment was replicated four times with four independent groups of 10 males and 10 females. Behavioral acts for each focal male were analyzed successively from the videotapes such that we could quantify what each focal animal in the group was doing at the same time. Several recordings were conducted repeatedly by the same observer and by different observers, and coding error was less than 4%.

### Part 2 – Effect of an audience on male behavior

**Figure 1:**
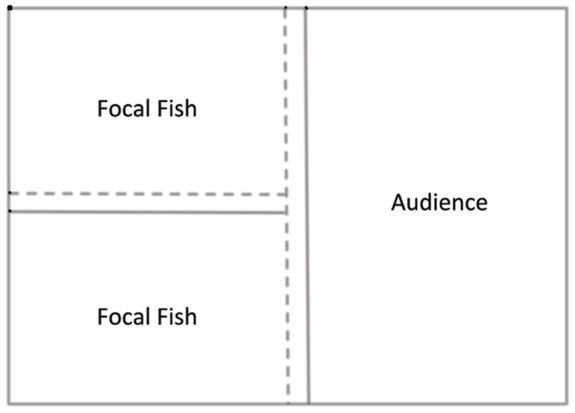
Experimental aquarium setup. Looking into the aquarium from directly above, focal fish would be in smaller adjacent compartments (equipped with a flowerpot shelter – not shown) while the audience would be in the larger compartment. Solid lines represent removable opaque barriers and dashed lines are permanent clear barriers, making the compartments water tight.

To test for effects on levels of aggressive displays in the presence of different audiences, pairs of size matched T (dominant) males were placed in adjacent compartments of a square aquarium (see Figure 1), subdivided with both clear permanent and opaque removable barriers. These “focal fish” (N = 30) interacted aggressively throughout the experiments. In a third compartment we housed the “audience” that were used to test the effect of observers on aggression in the focal fish. Each compartment of the aquarium was watertight, eliminating any olfactory communication between the fish. Compartments were also equipped with a gravel substrate and a half clay flowerpot for shelter and around which fish could establish a territory. The focal fish and the audience were allowed to acclimatize to and establish a territory in the experimental aquarium for three days. On the fourth day, opaque barriers were removed between the focal fish and the audience and the focal fish were allowed to interact for 20 minutes. These 20-minute interactions were videotaped and scored by two independent observers blind to the predictions of the experiment. Because behavior of individuals within a dyadic interaction are highly dependent on each other, aggressive behavior of only 1 of the focal fish was used in analysis. Regardless of which fish was used in the analysis, the overall trends in the outcome were identical therefore, the individual included in the analysis was randomized based on the locations within the tank. Between each set of experimental trials, the tanks were drained of all water and refilled with fresh aquarium water. Focal fish were tested on 5 audiences and one control: 1) a dominant male that was four times larger than the focal males, 2) a dominant male that was half the size of the focal male, 3) a dominant male that was size-matched to the focal male, 4) a group of 10 females of all reproductive states, 5) a single gravid (ripe with eggs) female and 6) the control where no audience was present and all opaque barriers were removed to control for possible increased aggression as a result of an adjacent, empty territory. There was an average of 2.5 weeks between the sets of trials. This amount of time is enough so that there is no carry over between trials. Once the videos were scored, behaviors were grouped based on whether they were overtly aggressive acts (biting and ramming) or whether they were aggressive displays (lateral displays and gill flaring). Overt aggression and aggressive displays were compared to scores from the control trials.

### Statistical analysis

This work was conducted in 2 separate experiments. In experiment 1, to determine the time course of behavior between individuals, pairwise cross-correlations were conducted between all individuals within each tank replicate. To determine whether subordinate individuals behaved more aggressively when dominant fish were in view or out of view and to determine against whom dominant males were aggressive, Mann-Whitney non-parametric tests for differences between groups were conducted on 18 individuals because data did not meet the assumptions of parametric tests.

In experiment 2, to determine whether the presence of an audience influenced aggressive behavior between focal males (N = 15), a single repeated measures ANOVA was conducted with display type (overt, display) as an independent variable. Subsequent post hoc repeated measures ANOVAs and Tukey's tests were conducted keeping the overall alpha level at 0.05.

## Results

### Part 1 – Assessing attention hierarchies

**Figure 2:**
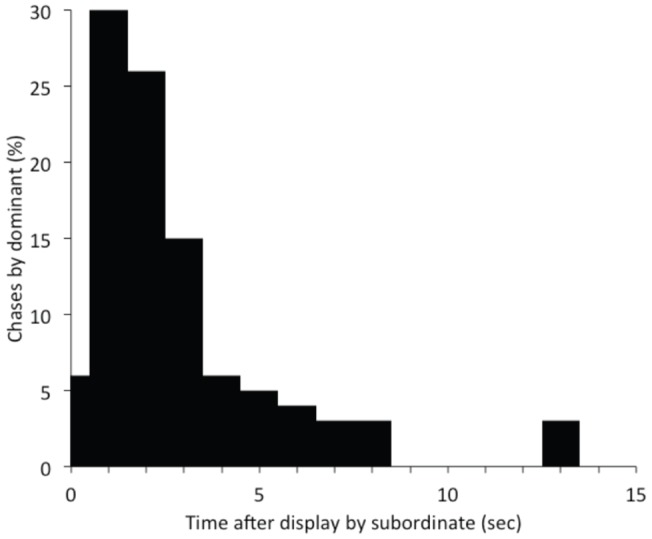
Aggression displayed while dominant male present and absent. Comparison of the number of agonistic, courtship and submissive behaviors performed by subordinates as a function of dominant male presence vs. absence. Chases directed against males (t = −6.617, p = 0.007) and females (t = −4.098, p = 0.033) as well as fleeing (t = 8.397, p = 0.004) were significantly different.

**Figure 3:**
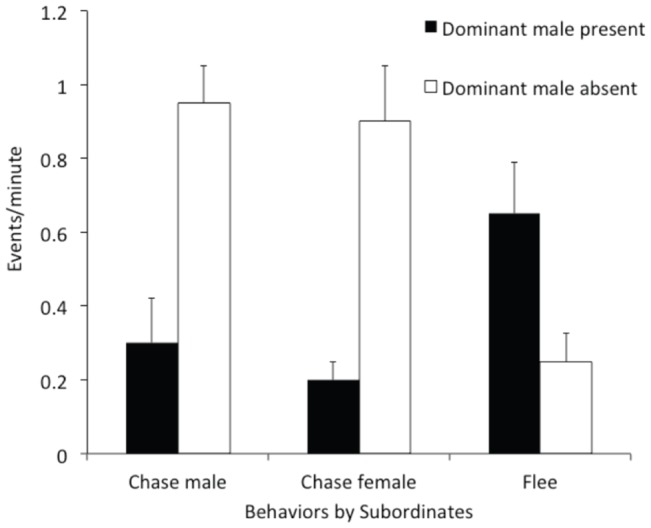
Aggressive displays by dominants relative to displays by subordinates. Aggressive displays by subordinate males elicit undirected responses by the dominant male. Most chases by the dominant occur within three seconds after an aggressive display by a subordinate.

We first asked whether there was any relationship between behavioral activity in pairs of T and NT males. Cross-correlation analysis between T and NT males showed that NT males never behaved aggressively at the same time as the dominant male (cross-correlation coefficient at t = 0: −0.21; t = −6.0782, p<0.001). Behavioral analysis showed that aggressive behavior in NT males depended on whether the T male was visible to them, or not. We found that when the T male was out of view, NT fish behaved much more aggressively and also courted females, behaviors that rarely occurred when the T male could see the NT male (Figure 2). T males, upon exiting their shelters attacked other individuals within a few seconds of returning to the group from a shelter (Figure 3). T males did not, however, specifically target the fish that had engaged in the aggressive behavior when he was out of view. On average, T males were aggressive equally against NT males who had just performed an aggressive act as compared with those that had not (Mann-Whitney test: U = 44, n_1_ = 8, n_2_ = 10, p = 0.66).

### Part 2 – Effect of an audience on male behavior

**Figure 4:**
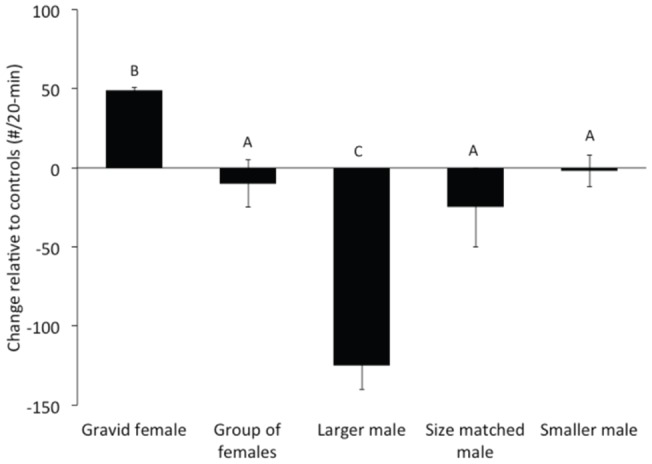
Overt aggressive acts. Number of overt aggressive acts (biting and ramming) displayed by signaling males in the presence of different audiences. The number of over aggressive acts varied across all audience types and the letters above the bars represent significant differences at α = 0.05. Differences in the number of aggressive acts performed in the presence of a group of females, size-matched males or smaller males did not differ from the control condition.

**Figure 5:**
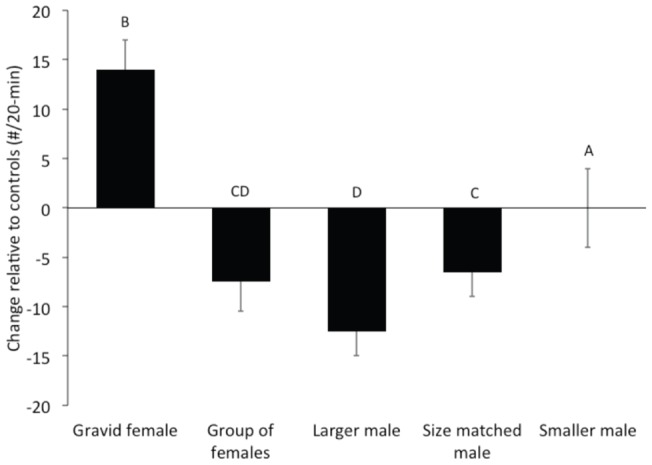
Aggressive displays and postures. Number of aggressive displays and postures by signaling males in the presence of different audiences. The number of over aggressive acts varied across all audience types and the letters above the bars represent significant differences at α = 0.05.

To test whether aggressive displays in *A. burtoni* depend on the nature of the audience, we observed fights between focal males in the presence of different audiences. Overall, there was a significant effect of audience type (F_1,28_ = 25.640, p<0.001) but there was no difference between display types (F_1,28_ = 0.008, p = 0.063). When fighting males were viewed by a larger, more dominant male, they decreased their number of overt aggressive interactions relative to controls (Figure 4; F_4,11_ = 8.045, p = 0.012) as well as their aggressive displays (Figure 5; F_4,11_ = 4.565, p = 0.03). In contrast, when focal males fought in the presence of a single gravid female, focal males not only increased their overt aggression (F_4,11_ = 5.342, p = 0.019) but also increased the number of aggressive postures, relative to controls (F_4,11_ = 2.05, p = 0.06), however this difference only approached significance. When fighting in the presence of an audience of a group of females (varying in reproductive state), a size matched or a smaller male, there was no difference in the number of overt aggressive behaviors (F_4,11_ = 0.386, p = 0.51) relative to controls. However, the number of aggressive postures decreased when signaling males were viewed by a group of females (F_4,11_ = 3.564, p = 0.04), or a size-matched male (F_4,11_ = 3.011, p = 0.05). When in the presence of an audience of a smaller male, there was no difference in aggressive postures (F_4,11_ = 0.632, p = 0.41).

## Discussion

Primates, including human children in social groups, attend carefully to the behavior of conspecifics during social interactions [20]. In some primate species, subordinates appear to continuously monitor the behavior of higher-ranking individuals [21]. These “attention hierarchies” oblige individual group members to attend to higher-ranking individuals and are important in preserving social group stability [7]. Here we report for the first time, the presence of attention hierarchies in fish. Attending to dominant males was observed in naturalistic conditions in stable social systems. As expected in an attention hierarchy, T and NT males essentially never behaved aggressively at the same time within the same social group. Thus the ongoing social behavior of NT males depends critically on their observation of the behavior of T males.

By recording whether T males were visible or not to NT males, we tested the hypothesis that some NT males would behave like T males when the T male was occupied or out of view. Indeed, with the T male out of view, NT males were significantly more likely to court females and act aggressively towards other fish. Since NT males modify their aggressive and courtship behaviors based on visibility of the T male, these animals are using social information to change their activities. In addition to the measurements of visibility, further evidence that the T and NT males were not in visual contact was the fact that T males did not specifically target those NT males that behaved aggressively or courted females when T males emerged from their shelter. Rather, they were equally aggressive towards all NT males, whether they had just been behaving dominantly, or not.

Modulating their behavior would potentially benefit the NT males by reducing the aggressive acts inflicted upon them by T males. Moreover, modification of behavior depending on conditions could signal reproductive opportunities with a subordinate male to females, even in the presence of a dominant male. Recently, it has been shown that *A. burtoni* NT males retain some reproductive function and can produce viable sperm [22]. In addition to maintaining reproductive opportunities, subordinates may also use times when a T male is not visible to establish or maintain their position relative to other subordinate males. Establishing and maintaining position within NT males may increase their changes for ascent in the dominance hierarchy, should the opportunity arise.

In *A. burtoni* it has been shown that turnover in the dominance hierarchy is common both in the wild [15] and in the laboratory [23] and that NT males very quickly assume dominant status and behaviors upon the removal of a T male [16], [17]. Thus the rapid modulation of behavior depending on the current social situation we have demonstrated here could be socially beneficial.

When fighting in the presence of an audience, males either increased or decreased their overt aggressive acts and their aggressive postures, based on the composition of the audience. Aggressive interactions between animals are potentially costly not only because of their energetic demands but also because of the increased risk of predation, serious injury or death. Consequently, animals typically assess opponents without direct combat, depending on reliable signals of aggressive intent [24] or of competitive ability [25], [26], [27], [28]. Signals used for assessment of competitive ability are thought to be honest because they depend on competitive ability. A corollary to this honest assessment is that animals of low competitive ability cannot produce signals characteristic of animals with high competitive ability [27], [28], [29]. We found that males fighting in the presence of a more dominant male decreased their level of aggression. By decreasing their aggressive displays, the focal males may be leading the larger (and likely more dominant male) in the audience to infer that the displaying males (and neighbors) do not pose a threat to reproductive opportunities. In the presence of a single gravid female, fighting males increased their level of aggressive. In this case, the females might infer that the fighting males are more dominant than they really are, which could result in females having their eggs fertilized by a less competitive male. The displaying male in both of these situations – either decreasing aggression in the presence of a larger male or increasing aggression in the presence of a gravid female – could thus be strategically minimize incurred aggression and maximize reproductive potential.

The displays examined here are within the normal repertoire of the signaler but could be interpreted as a form of social manipulation, causing the observer to possibly misinterpret what the acts signify, to the advantage of the signaler [30]. This type of social manipulation has been reported in a number of vertebrates [30], [31], [32], [33] including teleost fish [6], [34], [35], [36]. Pinto et al. [37] showed that bystanders avoid cleaners that they have witnessed cheating. In this case, image scoring by an audience leads to increased levels of cooperation [37]. Plath et al. [34] showed that mollies (*Poecilia mexicanus)*, reduced their courtship towards females to minimize the interception of information. Males directed their initial sexual interactions towards a non-preferred female when in the presence of a competitor that might induce surrounding males to copy the focal male's mate choice [34]. In this context, male mollies actively attend to the social environment and manipulate events for their own reproductive gains.

In humans and other primates, social context influences the extent to which subordinate animals express learned abilities [38]. For example, in rhesus macaques, subordinates who have learned an associative task, underperform this task in the presence of dominant males [39]. In *A. burtoni*, complex social relationships are sustained through strict attention structures. By closely attending to T males, NT males may conceal information from Ts while revealing their internal reproductive states to females. Plasticity in displays and the manipulation of others within a social group could translate into increased mating opportunities in NT males that otherwise do not have direct access to reproductive females but have the capacity to fertilize their eggs.
